# Day-to-Day Mobility, Affect, and Stress Couplings in Swiss Older Adults

**DOI:** 10.1093/geroni/igab046.1807

**Published:** 2021-12-17

**Authors:** Christina Roecke, Eun-Kyeong Kim, Pascal Griffel, Robert Moulder, Cheng Fu, Minxia Luo, Mike Martin, Robert Weibel

**Affiliations:** University of Zurich, Zurich, Zurich, Switzerland, 2 University of Zurich, Zurich, Zurich, Switzerland

## Abstract

The Mobility, Activity, and Social Interactions Study (MOASIS) is part of a global effort to more closely examine indicators of functional ability in relation to person characteristics and life contexts as proposed by the WHO’s healthy aging definition. In MOASIS, sensor-based and self-reported mobility and activity indicators were used to capture functional ability in 153 community-dwelling older adults aged 65-91 over 30 days. The present study examines daily time out-of-home and place diversity and its within-person associations with positive and negative affect and stress. Initial between-person analyses indicate that mobility is only weakly related to indicators of physical and mental health. We propose that the health- and well-being implications of mobility more strongly play out in daily life and at the within-person level, and will examine general health, cognitive ability, and marital status as intrinsic capacity moderators accounting for some of the expected interindividual heterogeneity.

